# HER2-specific recombinant immunotoxin 4D5scFv-PE40 passes through retrograde trafficking route and forces cells to enter apoptosis

**DOI:** 10.18632/oncotarget.15833

**Published:** 2017-03-02

**Authors:** Evgeniya Sokolova, Evgeniy Guryev, Andrey Yudintsev, Vladimir Vodeneev, Sergey Deyev, Irina Balalaeva

**Affiliations:** ^1^ Institute of Biology and Biomedicine, Lobachevsky University, Nizhny Novgorod 603950, Russia; ^2^ Institute of Regenerative Medicine, I.M. Sechenov First Moscow State Medical University, Moscow 119991, Russia; ^3^ Institute of Bioorganic Chemistry of the Russian Academy of Sciences, Moscow 117997, Russia

**Keywords:** targeted therapy, HER2, 4D5scFv, Pseudomonas aeruginosa exotoxin A, PE40

## Abstract

Immunotoxin 4D5scFv-PE40 is a recombinant protein that comprises 4D5scFv antibody as a targeting module and fragment of *Pseudomonas* exotoxin A as an effector (toxic) one. The immunotoxin has shown pronounced antitumor effect on cancer cells overexpressing HER2 receptor *in vitro* and on HER2-positive experimental tumors *in vivo*. We clarified the mechanism of 4D5scFv-PE40 activity that is of particular importance in the case of targeted therapeutic agent aimed at personalizing treatment of disease in relation to molecular genetic characteristics of each patient. After specific binding to HER2 on the cell surface and clathrin-mediated endocytosis the immunotoxin passes through retrograde trafficking route. During this route the immunotoxin molecule is supposed to undergo enzymatic processing that ends in separation of C-terminal and N-terminal fragments of the immunotoxin. Finally, C-terminal functionally active fragment of 4D5scFv-PE40 arrests protein synthesis in cytoplasm followed by cell death via apoptosis.

## INTRODUCTION

The basis of targeted therapy is precise elimination of tumor cells in the body while minimizing systemic toxicity. This is achieved through the use of drugs of directed action that are able to recognize tumor-specific antigens, or targets. The HER2 receptor is considered to be an effective molecular target for targeted therapy of several epithelial solid tumors due to significant differences in its expression level in normal and tumor cells. To date, four drugs are approved for targeted therapy of HER2-positive breast cancer: two monoclonal antibodies (trastuzumab and pertuzumab), low molecular weight reversible tyrosine kinase inhibitor (lapatinib) and antibody-drug conjugate (ADC) composed of monoclonal antibody and maytansinoid microtubule assembly inhibitor (trastuzumab emtansine) [1–4].

The last-named presents the most up-to-date strategy to create bifunctional constructs on the basis of antibody (or other targeting molecules) that include a powerful toxic module [5]. In such constructs antibody provides addressing, i.e. targeted delivery of the agent to tumor cells. Despite wide-spread occurrence of ADC technology, production and purification of chemical conjugates is time-consuming and technically complex process due to heterogeneity of derived constructs, complexity of their synthesis and frequent loss of functional properties of constituent modules. An alternative technology is creation of recombinant immunotoxins based on targeting and toxic modules connected by means of genetic engineering. Compared to chemical conjugation, this method allows to produce the necessary amount of functionally active molecules with strictly controlled composition [6].

We have previously created recombinant protein 4D5scFv-PE40 [7]. The targeting module of this immunotoxin is scFv fragment of the monoclonal HER2-specific antibody trastuzumab (Herceptin) [8]. A fragment of *Pseudomonas* exotoxin A (PE) was used as a toxic module of 4D5scFv-PE40. PE is a highly toxic protein that irreversibly inhibits protein synthesis in eukaryotic cells by ADP-ribosylation of the translation elongation factor 2 (eEF2) [9]. The PE fragment (PE40) lacks natural PE binding domain I and so comprises domains II, Ib and III of the wild-type PE. The PE40 module has KDEL sequence at its C-terminus that is a modification of the natural REDL sequence of wild-type PE [10]. This sequence is known to be the ER-retention signal ensuring retrograde transport of proteins to ER through specificity to KDEL receptor, that is a key process in wild-type PE productive intracellular trafficking [36–38]. Targeting and toxic modules are connected via flexible 16-aa linker from the mouse IgG_3_ hinge region [11]. The 4D5scFv-PE40 immunotoxin was shown to have selective cytotoxicity against HER2-positive cells with IC_50_ values in the low picomolar range, which are several orders of magnitude below the IC_50_ values for HER2-negative cells [7, 12]. Preliminary *in vivo* study revealed a pronounced antitumor effect of 4D5scFv-PE40 against HER2-overexpressing human ovarian carcinoma xenograft: a single injection of the immunotoxin in a dose of 4 mg per animal inhibited experimental tumors growth by about 80% compared with the untreated control [12].

Until new drug is introduced into clinical practice it is necessary to study the mechanisms of its action on the cellular level, since it opens the door for predicting pharmacodynamics, efficacy and safety in the organism. In this paper we show mechanism of the 4D5scFv-PE40 internalization and retrograde transport in the cell that allow the most of the immunotoxin molecules to remain functionally active. This results in protein synthesis arrest by 4D5scFv-PE40 and subsequent cell death via apoptosis.

## RESULTS

### 4D5scFv-PE40 binding specificity

**Figure 1:**
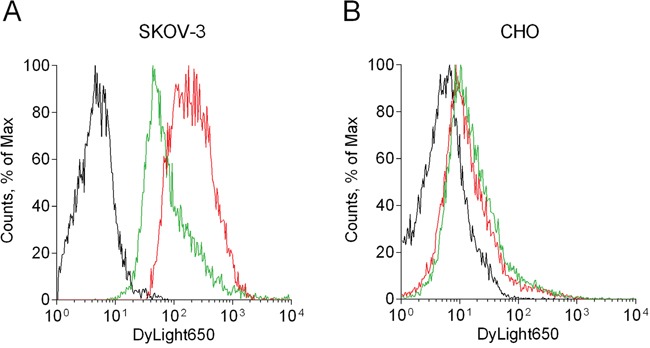
Study of the 4D5scFv-PE40 binding specificity Flow cytometry analysis of SKOV-3 **(A)** and CHO **(B)** cells after 1 h incubation at room temperature with 100 nM DyLight650-labeled 4D5scFv-PE40 alone (red line) or in the presence of equimolar amount of free 4D5scFv (green line). Black line represents control cells (autofluorescence).

It was shown previously that the 4D5scFv-PE40 immunotoxin based on HER2-specific 4D5scFv antibody as targeting module and 40 kDa fragment of *Pseudomonas aeruginosa* exotoxin A as toxic module binds with high affinity to recombinant extracellular domain of the HER2 receptor (Kd∼6.8 nM) [12]. In order to confirm the specificity of the immunotoxin binding to the HER2 receptor on cells the competitive binding assay was carried out using free 4D5scFv protein as a competitor for the HER2 epitope. Flow cytometry analysis showed that DyLight650-labeled 4D5scFv-PE40 alone effectively binds to HER2-overexpressing SKOV-3 cells (Figure 1A, red line with geometric mean of 176.8 vs. black autofluorescence line with geometric mean of 5.8). At the same time, co-incubation with 4D5scFv in equimolar concentration decreases the intensity of DyLight650 fluorescence more than twofold (Figure 1A, green line with geometric mean of 77.4). For HER2-negative CHO cells negligible and non-specific 4D5scFv-PE40 binding is showed (Figure 1B, red and green lines with geometric mean of 11.7 and 15.4, respectively, vs. black autofluorescence line with geometric mean of 5.7).

### 4D5scFv-PE40 internalization

**Figure 2:**
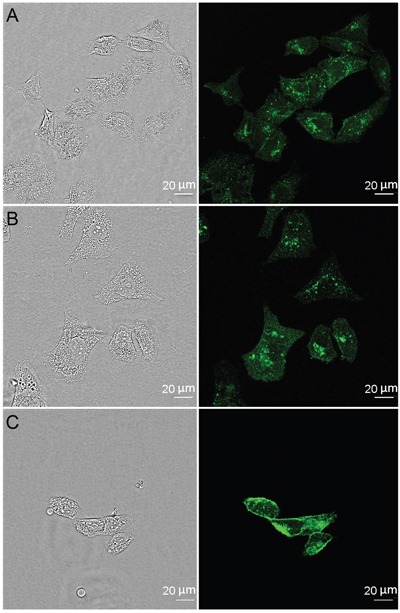
Internalization of 4D5scFv-PE40 SKOV-3 cells were treated with FITC-labeled 4D5scFv-PE40 for 2 h at 37 °C alone **(A)** or in the presence of 5 μg/ml filipin **(B)** or 5 μg/ml chlorpromazine **(C)** (see Materials and Methods section for details).

The mechanism of the 4D5scFv-PE40 internalization was studied by inhibition analysis of endocytosis pathways. SKOV-3 cells were incubated with FITC-labeled 4D5scFv-PE40 alone or in the presence of inhibitors of different endocytosis pathways. Chlorpromazine known to retain clathrin coats on endosomal membranes thus removing coated pits from cell surface [13] was used as clathrin-mediated endocytosis inhibitor, and filipin that disassembles caveolae by removing cholesterol from the plasma membrane [14, 15] was used as caveolae-mediated endocytosis inhibitor. Confocal microscopy images show that FITC-labeled immunotoxin distributes within target cell in the absence of inhibitors as well as in the presence of filipin (Figure 2A, 2B, respectively). Alternatively, chlorpromazine significantly slows down the 4D5scFv-PE40 internalization that is evident by pronounced FITC staining on cell membrane (Figure 2C). This clearly indicates that 4D5scFv-PE40 internalizes into target cell via clathrin-mediated endocytosis after binding HER2 at the cell surface.

### 4D5scFv-PE40 intracellular localization

**Figure 3:**
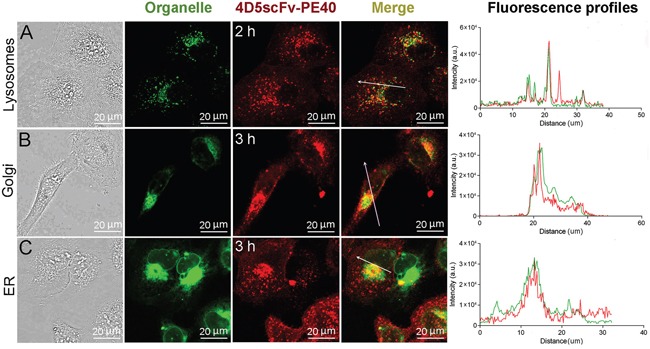
Intracellular localization of 4D5scFv-PE40 SKOV-3 cells were incubated with DyLight650-labeled 4D5scFv-PE40 and stained with LysoTracker Green after 2 h incubation **(A)**, Bodipy FL C5-ceramide after 3 h incubation **(B)** or ER-Tracker Green after 3 h incubation **(C)**. Fluorescence intensity profiles (right column) show the fluorescence intensity of DyLight650 and organelle dye along the randomly positioned arrow.

To visualize the 4D5scFv-PE40 intracellular pathway and make sure whether it is effective for PE40 delivery for further function, intracellular localization of DyLight650-labeled 4D5scFv-PE40 was analyzed in live SKOV-3 cells after fluorescent staining of organelles of interest. As shown in Figure 3A, 4D5scFv-PE40 localizes in lysosomes after 2 h incubation. Later 4D5scFv-PE40 is observed in Golgi apparatus (Figure 3B) as well as in ER (Figure 3C). The presented results suggest that the internalized immunotoxin partly undergoes lysosomal degradation. However, a considerable amount of 4D5scFv-PE40 molecules gets into ER that is probably provided by the KDEL sequence located at its C-terminus [10].

### 4D5scFv-PE40-induced protein synthesis inhibition

**Figure 4:**
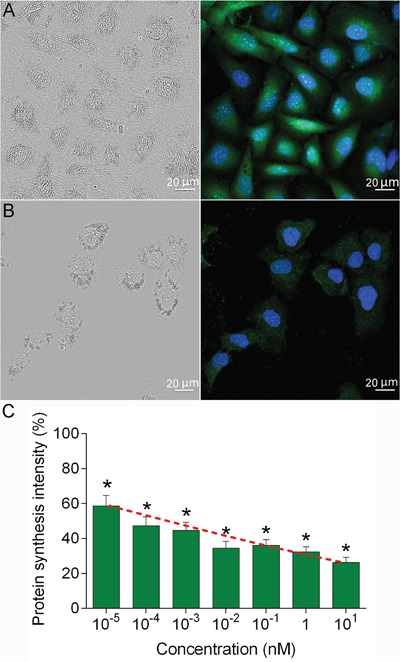
Protein biosynthesis inhibition induced by 4D5scFv-PE40 SKOV-3 cells were incubated with 4D5scFv-PE40 in different concentrations for 24 h, then the protein synthesis was assessed with Click-iT AHA Alexa Fluor 488 Protein Synthesis HCS Assay: confocal microscopy images of control cells **(A)** and cells treated with 1 nM 4D5scFv-PE40 **(B)**; protein synthesis intensity after 24 h incubation with different concentrations of 4D5scFv-PE40 **(C)**. The data are represented as mean ±SEM. * - p<0.0001 as compared to untreated control (Dannet's test, n>20). Red dashed curve represents data approximation with four-parameter dose-response curve (4PL).

The 4D5scFv-PE40 immunotoxin was previously shown to possess very high and selective cytotoxicity against HER2-expressing cells with IC_50_ values in low picomolar range [12]. To clarify the mechanism of this cytotoxic effect the level of protein biosynthesis in SKOV-3 cells was measured using Click-iT AHA Alexa Fluor 488 Protein Synthesis HCS Assay (Invitrogen) that provides labeling of newly synthesized proteins. It was shown that 24 h treatment of SKOV-3 cells with 4D5scFv-PE40 resulted in strong inhibition of protein biosynthesis (Figure 4B) as compared to untreated control cells (Figure 4A). This effect was observed in a dose-dependent manner with IC_50_ value of 0.05 pM (Figure 4C). Thus, the growth inhibition effect of 4D5scFv-PE40 is based on protein synthesis arrest in target cell.

### 4D5scFv-PE40-induced apoptosis

Wild-type PE-induced protein synthesis inhibition was shown to promote apoptosis in target cell [16–19]. In order to prove this for the 4D5scFv-PE40 immunotoxin the mechanism of cell death was evaluated.

**Figure 5:**
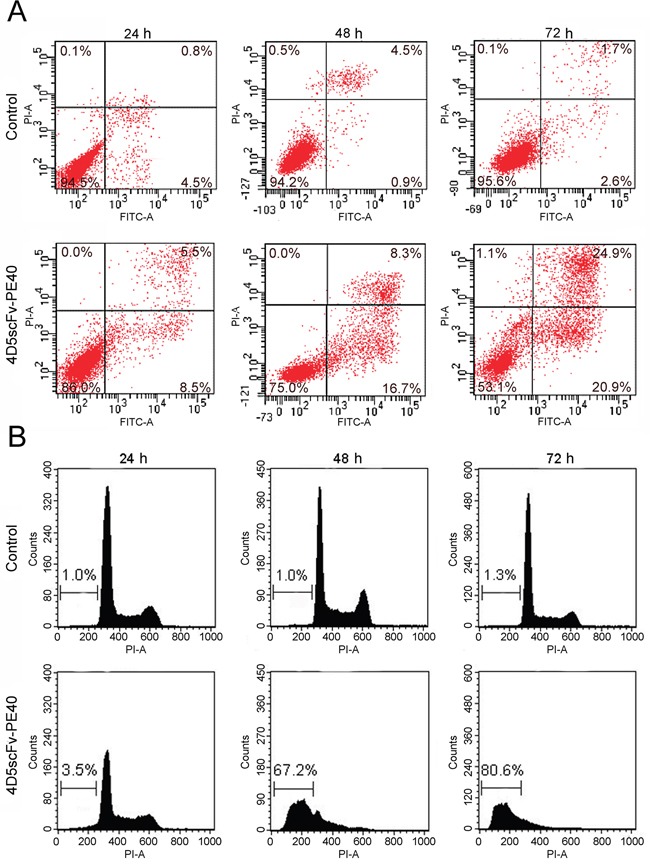
Induction of apoptosis in SKOV-3 cells after treatment with 4D5scFv-PE40 **(A)** flow cytometry analysis of phosphatidylserine exposure after incubation with 50 nM 4D5scFv-PE40 for 24, 48 or 72 h; **(B)** flow cytometry analysis of hypoploid sub-G1 nuclei after incubation with 50 nM 4D5scFv-PE40 for 24, 48 or 72 h.

One of the earliest features of apoptosis is loss of plasma membrane lipid asymmetry and translocation of phosphatidylserine from the inner to the outer leaflet of the membrane. Annexin V-FITC and PI staining of SKOV-3 cells incubated with 4D5scFv-PE40 revealed pronounced phosphatidylserine externalization (Figure 5A). The percent of cells undergoing apoptosis, e.g. both early apoptotic cells (PI negative, Annexin V-FITC positive) and late apoptotic cells (PI positive, Annexin V-FITC positive) reached 46% after 72 h treatment with 4D5scFv-PE40.

Induction of apoptosis in 4D5scFv-PE40-treated SKOV-3 cells was also confirmed by nuclear fragmentation analysis. SubG1 population rate was substantially increased in cells treated with 50 nM 4D5scFv-PE40 from 3.5% to 80% during 72 h incubation (Figure 5B). It should be noted that DyLight650-labeled derivative caused the same rate of nuclear fragmentation as compared with unlabeled immunotoxin (Supplementary Figure 2) suggesting that labeling does not modify the intracellular route and activity of the immunotoxin.

These results suggest that the observed cytotoxic effect of 4D5scFv-PE40 on HER2-overexpressing cells is mediated through an apoptotic mechanism.

## DISCUSSION

Upregulated expression of the HER2 receptor is common with a number of carcinomas of different localizations and considered to be an important prognostic criterion in clinic. At the same time the HER2 receptor itself has become a successfully approved molecular target for a targeted therapy. Despite the fact that the HER2-targeted therapy is based on monoclonal antibodies, there is a known issue of their insufficient effectiveness primarily because of acquired resistance of cancer cells to MAb therapy. Mechanisms of the resistance are various and reflect both branching of the HER2-mediated signaling system with possible activation of alternative pathways, and high genetic variability of cancer cells. For example, Trastuzumab-induced HER2 blocking may lead to activation of bypass signal pathways that are triggered by other receptor tyrosine kinases (insulin-like growth factor 1 receptor (IGF-1R), vascular endothelial growth factor receptor (VEGFR), ect.) [20]. In this context “loading” of MAbs with additional toxic module has been strongly motivated and resulted in development of bifunctional targeted agents, such as immunotoxins [5, 21, 22].

Immunotoxin consists of an antibody or its recognizing fragment for addressing and an effector component that is highly toxic protein. The most widely used effector proteins are protein toxins of AB structure, i.e. consisting of two functionally independent subunits, catalytic one that blocks specific activity in cytoplasm (subunit A) and binding one that determines natural specificity of the toxin (subunit B). *Pseudomonas* exotoxin A presents an example of AB toxins and is composed of three major domains: domain Ia is receptor-specific, domain II mediates transport to cytoplasm, domain III catalyzes ADP-ribosylation and inactivation of eEF2 in the cytoplasm. Minor domain Ib located sequentially between domain II and III has been considered to contribute to catalytic activity of PE [23]. The intoxication pathway of wild-type PE is well studied. It is a sequence of events that precede the exit of its catalytic subunit into the cytoplasm: (i) binding of domain Ia to α2-macroglobuline receptor at the cell surface and internalization; (ii) proteolytic cleavage and reduction of a disulfide bond in domain II resulting in dissociation of A and B subunits; (iii) retrograde transport from endosomes to ER. It was shown previously that PE modifications or mutations in target cell that impair this multiple-stage pathway dramatically decrease PE cytotoxicity [24]. Thereby the effectiveness of targeted agents utilizing PE as effector compound depends not only on selectivity toward target cells but also on proper intracellular performance of PE or its fragment.

In this study we ascertain the mechanism of action of recombinant immunotoxin 4D5scFv-PE40 that showed significant cytotoxic effect against HER2-overexpressing cells *in vitro* and *in vivo* in the pilot study [7, 12]. The immunotoxin combines HER2-specific antibody 4D5scFv and PE fragment (PE40) that lacks natural binding domain I and so comprises domains II, Ib and III of wild-type PE (252-612 a.a.). The natural C-terminal REDL sequence of wild-type PE was replaced with KDEL sequence [10] in order to improve its intracellular traffic and cytotoxicity.

The 4D5scFv antibody has been successfully used in our previous studies for tumor cell-targeted delivery of therapeutic [25–28] and diagnostic [29–31] agents of different nature both *in vitro* and *in vivo*. Here we show that 4D5scFv being a part of the recombinant immunotoxin retains its functionality and so provides addressed delivery of the PE fragment to HER2-overexpressing cells due to specific binding to the HER2 receptor (Figure 1).

Considering that cytotoxic effect of PE-based immunotoxins is realized in cytoplasm, their affine and selective binding to target receptor must be followed by endocytosis. It is known that HER2 internalizes with rather low efficiency as homodimers as well as heterodimers with other HER family members that accounts for its role in activation of signal transduction [32, 33]. It has also been shown that not all complexes of HER2 with specific monoclonal antibodies or artificial ligands undergo internalization [34] that may depend on a variety of factors such as target cell biology [35] and valency of ligand [36, 37]. In this context the internalization of HER2 targeted therapeutic agents should be addressed in every particular case. We demonstrate in this study that the 4D5scFv-PE40 immunotoxin does internalize into target cells and reveal that this process is clathrin-dependent (Figure 2). This clearly indicates that the 4D5scFv antibody provides the immunotoxin entering in cell via clathrin-mediated endocytosis after binding to HER2.

Somewhere along the intracellular route, the immunotoxin is supposed to undergo processing at domain II of PE40, just as it occurs for wild-type PE, that results in separation of C-terminal and N-terminal fragments of the immunotoxin. Analysis of intracellular localization testifies to productive transport of 4D5scFv-PE40 (or its C-terminal fragment only) to ER (Figure 3) that is apparently mediated by KDEL sequence at the C-terminus of the immunotoxin molecule [10]. The KDEL sequence is a modification of the natural REDL sequence of wild-type PE and is known to be a key in the process of retrograde transport of proteins in cell through specificity to KDEL receptor at Golgi membrane [38, 39]. It was previously shown that the PE fragment (subunit A) being transported in ER then translocates into cytoplasm via ER-associated protein degradation (ERAD) mechanism [24, 40]. So, it appears that 4D5scFv-PE40-derived PE fragment undergoes transport route in target cell similar to that of wild-type PE. We did not specify when the immunotoxin cleavage occurs during the pathway, however, the results obtained allow to conclude that its targeting module 4D5scFv does not affect the PE40 behavior in cell. This is in agreement with 4D5scFv-PE40-induced protein synthesis inhibition in target cells SKOV-3 (Figure 4) that corresponds to the wild-type PE cytotoxicity mechanism. The sum of these results gives a good reason to high cytotoxicity of 4D5scFv-PE40 against HER2-positive cells expressed in cell growth inhibition with IC_50_ values in the low picomolar range [12].

Type of induced cell death presents an important issue when looking forward to clinical application of anti-tumor agent. Apoptosis is considered to be the most preferable since it is not attended with inflammation unlike necrosis. At the same time apoptotic cell death may increase tumor immunogenicity and stimulate anti-tumor immune response [41]. A number of PE-based immunotoxins with different specificity was shown to drive apoptosis in target cells [42–46]. Here we also show induction of apoptosis in SKOV-3 cells as a result of 4D5scFv-PE40 treatment (Figure 5). However interrelation between protein synthesis inhibition and apoptosis trigger remains to be clarified. Several studies consider these two processes as independent mechanisms leading to cell death under PE-based immunotoxins [47, 48]. On the other hand straight cause-effect link has been established between them. Thus, disrupting the balance between antagonist proteins of Bcl-2 family, pro-apoptotic Bak and short-lived anti-apoptotic Mcl-1, was shown in response to PE-induced protein synthesis inhibition. This was suggested as critical trigger point for apoptosis in fibroblasts [49] as well as in breast carcinoma cells MA-11 [50]. The influence of expression level of anti-apoptotic protein Bcl-2 on effectiveness of apoptosis caused by PE-based immunotoxins was also reported for lymphoma cell lines and breast carcinoma cells MCF-7 [51, 52]. Probably, apoptosis regulator proteins may contribute to different extent to processes activated after protein synthesis inhibition that depends on the cell molecular profile. Interestingly, our results reveal remarkable time separation between protein synthesis arrest and appearance of early features of apoptosis (phosphatidylserine externalization). Thus, 24 h treatment of SKOV-3 cells with 4D5scFv-PE40 in nanomolar concentrations inhibited protein synthesis by more than 70% although only negligible apoptosis rate was observed at this time point. This fact likely reflects alterations of apoptotic machinery in SKOV-3 cells, particularly mutations in the p53 gene causing absence of p53 transcripts and protein [53]. Implication of some survival programs provided by increased expression of anti-apoptotic protein genes may also play part.

**Figure 6:**
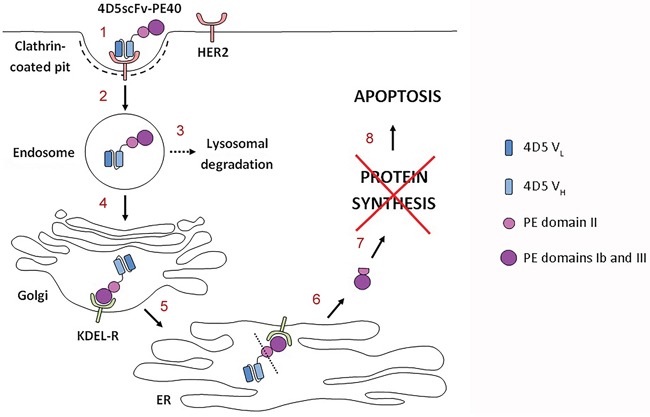
Proposed mechanism of the 4D5scFv-PE40 immunotoxin cytotoxicity After specific and affine binding of the immunotoxin to the HER2 receptor (**1**), it internalizes via clathrin-mediated endocytosis (**2**). The internalized immunotoxin undergoes both lysosomal degradation (**3**) and productive trafficking into Golgi apparatus (**4**). In Golgi apparatus the C-terminal KDEL sequence of the immunotoxin binds to the KDEL receptor (KDEL-R) that mediates the protein retrograde transport into endoplasmic reticulum (ER) (**5**). Somewhere along the transport pathway the immunotoxin molecule possibly undergoes enzymatic processing resulting in separation of functionally active PE fragment that comes out of ER into the cytoplasm (**6**) and inhibits protein synthesis there (**7**). Protein synthesis inhibition leads to apoptosis of target cell (**8**).

So, the effectiveness of targeted anti-tumor immunotoxin 4D5scFv-PE40 based on HER2-specific antibody 4D5scFv and PE40 that we have shown previously is caused by target cell apoptosis and determined by combination of factors: (i) specific and affine binding of the immunotoxin to the HER2 receptor; (ii) effective internalization of the “receptor-immunotoxin” complex via clathrin-mediated endocytosis and (iii) productive intracellular transport of the immunotoxin providing delivery of functionally active PE fragment into the cytoplasm (Figure 6).

In conclusion, our results prove high potential of HER2-specific recombinant immunotoxin 4D5scFv-PE40 as an agent for targeted therapy of HER2-positive tumors. The mechanism of the 4D5scFv-PE40 action presented here may (i) predetermine its pharmacodynamics; (ii) underlie its therapeutic efficacy and limitations thereof coming from alterations in cancer cell genome and proteome; (iii) predict its side effects. All of this gives the option to effective and safe use of the immunotoxin in clinical practice.

## MATERIALS AND METHODS

### 4D5scFv-PE40 expression and purification

The 4D5scFv-PE40 recombinant protein was expressed in *E.coli* BL21(DE3) using the pSD-4D5scFv-ETA plasmid and purified by metal chelate affinity and ion exchange chromatography as described in [12]. For details, please, see Supplementary Data.

### Labeling of 4D5scFv-PE40 with fluorescent dyes

4D5scFv-PE40 was labeled with fluorescein isothiocyanate (FITC, Thermo Scientific) or DyLight650 NHS Ester (Thermo Scientific), that are widely used amino-reactive dyes. These dyes contain isothiocyanate or N-hydroxysuccinimide (NHS) ester, respectively, reacting with primary amines in proteins. Prior to labeling reaction 4D5scFv-PE40 was desalted on Sephadex G-25 column (PD SpinTrap G-25, GE Healthcare) equilibrated with borate buffer (400 mM H_3_BO_3_, 70 mM Na_2_B_4_O_7_, pH 8.0). For FITC labeling, the protein was incubated with 5-fold molar excess of FITC dissolved in DMSO (Thermo Scientific) for 2 h at room temperature in the dark. For DyLight650 labeling, the protein was incubated with 7-fold molar excess of DyLight650 dissolved in DMSO (Thermo Scientific) for 1 h at room temperature in the dark. To remove unbound dye the reaction mixture was then desalted on Sephadex G-25 column (PD SpinTrap G-25, GE Healthcare) equilibrated with PBS.

### Cell lines

HER2-overexpressing SKOV-3 cells (human ovarian adenocarcinoma, ATCC number HTB-77) and HER2-negative CHO cells (Chinese hamster ovary, ATCC number CCL-61) were cultured in McCoy's 5A medium with 10% (v/v) fetal calf serum (HyClone) and 2 mM L-glutamine. Cells were grown in 5% CO_2_ at 37°C. For passaging cells were carefully detached using Versene solution (PanEco) in order to prevent proteolysis of membrane proteins. The level of HER2 expression in these cells was estimated by flow cytometry after staining of cell suspensions with HER2-specific mouse monoclonal antibodies conjugated with FITC (Supplementary Figure 1).

### Analysis of 4D5scFv-PE40 binding specificity

SKOV-3 and CHO cells were detached and incubated in PBS with 100 nM 4D5scFv-PE40 labeled with DyLight650 alone or in the presence of free 4D5scFv in equimolar concentration for 1 h at room temperature. Then cells were washed twice with PBS to remove unbound proteins and analyzed by flow cytometry using a FACSCalibur instrument (BD Biosciences) equipped with a 635 nm laser to excite DyLight650.

### Study of 4D5scFv-PE40 internalization

SKOV-3 cells were seeded in 96-well flat bottom plates at a density of 5×10^3^ cells per well and allowed to attach overnight. To analyze the pathway of 4D5scFv-PE40 internalization cells were pre-incubated with inhibitors of clathrin- or caveolae-mediated endocytosis: chlorpromazine (5μg/ml) or filipin (5μg/ml) (Sigma), respectively, in growth conditions for 30 min. After that cells were incubated with 500 nM 4D5scFv-PE40 labeled with FITC in the presence of these inhibitors (5μg/ml) for 2 h in growth conditions, then washed thrice with PBS and fixed with 4% formaldehyde. Images were obtained by confocal microscopy using LSM 710 system (Carl Zeiss) with 40× water C-Apochromat objective with numerical aperture of 1.2. The 4D5scFv-PE40-FITC was excited by the 488 nm argon laser, the fluorescence was collected in the range of 505-600 nm.

### Analysis of 4D5scFv-PE40 intracellular localization

SKOV-3 cells were seeded in 96-well flat bottom plates at a density of 5×10^3^ cells per well and allowed to attach overnight. Then cells were incubated with DyLight650-labeled 4D5scFv-PE40 for different periods of time (up to 3 h), washed with PBS and stained with specific dyes for lysosomes (LysoTracker Green, Molecular probes), Golgi apparatus (Bodipy FL C5-ceramide, Molecular probes) and endoplasmic reticulum (ER-Tracker Green, Molecular probes) according to the manufacturer's instruction. Images were obtained by LSM 710 system (Carl Zeiss) with 40× water C-Apochromat objective with numerical aperture of 1.2. The 4D5scFv-PE40-DyLight650 was excited by the 635 nm HeNe laser, the fluorescence was collected in the range of 643-735 nm. The organelle probe dyes were excited by the 488 nm argon laser, the fluorescence was collected in the range of 509-566 nm.

### Protein inhibition assay

SKOV-3 cells were seeded in 96-well flat bottom plates at a density of 5×10^3^ cells per well and allowed to attach overnight. The medium was then exchanged for fresh growth medium containing different concentrations of 4D5scFv-PE40 and cells were incubated for 24 h at 37°C in 5% CO_2_. The protein synthesis was then assessed using Click-iT AHA Alexa Fluor 488 Protein Synthesis HCS Assay (Molecular Probes) according to the manufacturer's instruction. This assay is based on incorporation of methionine analog containing an azide moiety (L-azidohomoalanine) into proteins during protein synthesis in cells incubated in methionine-free growth medium. Then the incorporated L-azidohomoalanine is detected with fluorescent labeling via chemoselective click-reaction between an azide and alkyne (in this case, Alexa Fluor 488 alkyne). So, Alexa Fluor 488 fluorescence intensity in the cell cytoplasm correlates with the protein synthesis activity. Images of cells were obtained by LSM 710 system (Carl Zeiss) with 40× water C-Apochromat objective with numerical aperture of 1.2. The Alexa Fluor 488 was excited by the 488 nm argon laser, the fluorescence was collected in the range of 507-585 nm. For visualization of cell nuclei counterstained with Hoechst33342 the 750 nm Ti:Sapphire femtosecond laser was used for two-photon excitation, the fluorescence was collected in the range of 415-498 nm. The protein synthesis was measured by determining integral signal intensity of Alexa Fluor 488 within the cytoplasm area except autofluorescence (Carl Zeiss ZEN 2 blue edition). Data are presented as mean ± SEM. Statistical analysis was performed using one-way ANOVA and Dunnett's test (GraphPad Prism 6 software).

### Detection of phosphatidylserine exposure

SKOV-3 cells were seeded in 6-well plates at a density of 2×10^5^ cells per well and allowed to attach overnight. The medium then was exchanged for fresh growth medium containing 50 nM 4D5scFv-PE40 and cells were incubated for 24, 48 or 72 h at 37°C in 5% CO_2_. To detect phosphatidylserine exposure in cells with integral plasma membrane SKOV-3 cells were then stained with FITC Annexin V Apoptosis Detection Kit I (BD Pharmingen) containing Annexin V-FITC and propidium iodide (PI): the former is FITC-labeled protein that specifically binds to phosphatidylserine, and the latter is DNA dye that permeates only cells with damaged membrane (dead cells). After this staining four populations of cells can be identified by flow cytometry that we have interpreted as: viable cells (PI and Annexin V-FITC negative), early apoptotic cells (PI negative, Annexin V-FITC positive), late apoptotic and dead cells (PI positive, Annexin V-FITC positive) and cells died via non-apoptotic process (PI positive, Annexin V-FITC negative). Flow cytometry was carried out using FACSAria III instrument (BD) equipped with a 488 nm laser to excite FITC and propidium iodide (PI).

Data collection and analysis was performed using FACSDiva software (BD).

### Detection of hypoploid nuclei

SKOV-3 cells were seeded in 6-well plates at a density of 5×10^5^ cells per well and allowed to attach overnight. The medium then was exchanged for fresh growth medium containing 50 nM 4D5scFv-PE40 and cells were incubated for 24, 48 or 72 h at 37°C in 5% CO_2_. After that cells were analyzed with CycleTest Plus DNA reagent Kit (BD Pharmingen) that involves cell nuclei isolation and PI staining. Flow cytometry analysis of PI fluorescence indicates the nuclear DNA content thus identifying cell-cycle phase distributions: 2c nuclear DNA content in G_1_/G_0_ phase, DNA contents ranging from 2c to 4c in S phase, and 4c nuclear DNA content in G_2_/M phase. Nuclear fragments with DNA content less than 2c resulted from apoptosis (hypoploid nuclei) fall into sub-G_1_ region. The cell nuclei were analyzed using FACSCalibur cytometer (BD) equipped with a 488 nm laser to excite PI.

Data collection and analysis was performed using CellQuest Pro software (BD).

## SUPPLEMENTARY MATERIALS FIGURES



## References

[1] Valabrega G, Montemurro F, Aglietta M (2007). Trastuzumab: mechanism of action, resistance and future perspectives in HER2-overexpressing breast cancer. Ann Oncol.

[2] Amiri-Kordestani L, Wedam S, Zhang L, Tang S, Tilley A, Ibrahim A, Justice R, Pazdur R, Cortazar P (2014). First FDA approval of neoadjuvant therapy for breast cancer: pertuzumab for the treatment of patients with HER2-positive breast cancer. Clin Cancer Res.

[3] Lambert JM, Chari RV (2014). Ado-trastuzumab Emtansine (T-DM1): an antibody-drug conjugate (ADC) for HER2-positive breast cancer. J Med Chem.

[4] Tevaarwerk AJ, Kolesar JM (2009). Lapatinib: a small-molecule inhibitor of epidermal growth factor receptor and human epidermal growth factor receptor-2 tyrosine kinases used in the treatment of breast cancer. Clin Ther.

[5] Weiner GJ (2015). Building better monoclonal antibody-based therapeutics. Nat Rev Cancer.

[6] Choudhary S, Mathew M, Verma RS (2011). Therapeutic potential of anticancer immunotoxins. Drug Discov Today.

[7] Sokolova EA, Zdobnova TA, Stremovskiy OA, Balalaeva IV, Deyev SM (2014). Novel recombinant anti-HER2/neu immunotoxin: design and antitumor efficiency. Biochemistry (Mosc).

[8] Eigenbrot C, Randal M, Presta L, Carter P, Kossiakoff AA (1993). X-ray structures of the antigen-binding domains from three variants of humanized anti-p185HER2 antibody 4D5 and comparison with molecular modeling. J Mol Biol.

[9] Wolf P, Elsasser-Beile U (2009). Pseudomonas exotoxin A: from virulence factor to anti-cancer agent. Int J Med Microbiol.

[10] Seetharam S, Chaudhary VK, FitzGerald D, Pastan I (1991). Increased cytotoxic activity of Pseudomonas exotoxin and two chimeric toxins ending in KDEL. J Biol Chem.

[11] Muller KM, Arndt KM, Strittmatter W, Pluckthun A (1998). The first constant domain (C(H)1 and C(L)) of an antibody used as heterodimerization domain for bispecific miniantibodies. FEBS Lett.

[12] Zdobnova T, Sokolova E, Stremovskiy O, Karpenko D, Telford W, Turchin I, Balalaeva I, Deyev S (2015). A novel far-red fluorescent xenograft model of ovarian carcinoma for preclinical evaluation of HER2-targeted immunotoxins. Oncotarget.

[13] Wang LH, Rothberg KG, Anderson RG (1993). Mis-assembly of clathrin lattices on endosomes reveals a regulatory switch for coated pit formation. J Cell Biol.

[14] Rothberg KG, Ying YS, Kamen BA, Anderson RG (1990). Cholesterol controls the clustering of the glycophospholipid-anchored membrane receptor for 5-methyltetrahydrofolate. J Cell Biol.

[15] Rothberg KG, Heuser JE, Donzell WC, Ying YS, Glenney JR, Anderson RG (1992). Caveolin, a protein component of caveolae membrane coats. Cell.

[16] Jenkins CE, Swiatoniowski A, Issekutz AC, Lin TJ (2004). Pseudomonas aeruginosa exotoxin A induces human mast cell apoptosis by a caspase-8 and -3-dependent mechanism. J Biol Chem.

[17] Sharma AK, FitzGerald D (2010). Pseudomonas exotoxin kills Drosophila S2 cells via apoptosis. Toxicon.

[18] Komatsu N, Oda T, Muramatsu T (1998). Involvement of both caspase-like proteases and serine proteases in apoptotic cell death induced by ricin, modeccin, diphtheria toxin, and pseudomonas toxin. J Biochem.

[19] Kochi SK, Collier RJ (1993). DNA fragmentation and cytolysis in U937 cells treated with diphtheria toxin or other inhibitors of protein synthesis. Exp Cell Res.

[20] Fiszman GL, Jasnis MA (2011). Molecular Mechanisms of Trastuzumab Resistance in HER2 Overexpressing Breast Cancer. Int J Breast Cancer.

[21] Sapra P, Shor B (2013). Monoclonal antibody-based therapies in cancer: advances and challenges. Pharmacol Ther.

[22] Pastan I, Hassan R, FitzGerald DJ, Kreitman RJ (2007). Immunotoxin treatment of cancer. Annu Rev Med.

[23] Kreitman RJ (2006). Immunotoxins for targeted cancer therapy. Aaps J.

[24] Weldon JE, Pastan I (2011). A guide to taming a toxin--recombinant immunotoxins constructed from Pseudomonas exotoxin A for the treatment of cancer. Febs J.

[25] Edelweiss E, Balandin TG, Ivanova JL, Lutsenko GV, Leonova OG, Popenko VI, Sapozhnikov AM, Deyev SM (2008). Barnase as a new therapeutic agent triggering apoptosis in human cancer cells. PLoS One.

[26] Balandin TG, Edelweiss E, Andronova NV, Treshalina EM, Sapozhnikov AM, Deyev SM (2011). Antitumor activity and toxicity of anti-HER2 immunoRNase scFv 4D5-dibarnase in mice bearing human breast cancer xenografts. Invest New Drugs.

[27] Serebrovskaya EO, Edelweiss EF, Stremovskiy OA, Lukyanov KA, Chudakov DM, Deyev SM (2009). Targeting cancer cells by using an antireceptor antibody-photosensitizer fusion protein. Proc Natl Acad Sci U S A.

[28] Mironova KE, Proshkina GM, Ryabova AV, Stremovskiy OA, Lukyanov SA, Petrov RV, Deyev SM (2013). Genetically encoded immunophotosensitizer 4D5scFv-miniSOG is a highly selective agent for targeted photokilling of tumor cells in vitro. Theranostics.

[29] Balalaeva IV, Zdobnova TA, Krutova IV, Brilkina AA, Lebedenko EN, Deyev SM (2012). Passive and active targeting of quantum dots for whole-body fluorescence imaging of breast cancer xenografts. J Biophotonics.

[30] Balalaeva IV, Zdobnova TA, Sokolova EA, Deyev SM (2015). [Targeted Delivery of Quantum Dots to HER2-Expressing Tumor Using Recombinant Antibodies]. Bioorg Khim.

[31] Zdobnova TA, Stremovskiy OA, Lebedenko EN, Deyev SM (2012). Self-assembling complexes of quantum dots and scFv antibodies for cancer cell targeting and imaging. PLoS One.

[32] Sorkin A, Di Fiore PP, Carpenter G (1993). The carboxyl terminus of epidermal growth factor receptor/erbB-2 chimerae is internalization impaired. Oncogene.

[33] Harari D, Yarden Y (2000). Molecular mechanisms underlying ErbB2/HER2 action in breast cancer. Oncogene.

[34] Rubin I, Yarden Y (2001). The basic biology of HER2. Ann Oncol.

[35] Hashizume T, Fukuda T, Nagaoka T, Tada H, Yamada H, Watanabe K, Salomon DS, Seno M (2008). Cell type dependent endocytic internalization of ErbB2 with an artificial peptide ligand that binds to ErbB2. Cell Biol Int.

[36] Guillemard V, Nedev HN, Berezov A, Murali R, Saragovi HU (2005). HER2-mediated internalization of a targeted prodrug cytotoxic conjugate is dependent on the valency of the targeting ligand. DNA Cell Biol.

[37] Vaidyanath A, Hashizume T, Nagaoka T, Takeyasu N, Satoh H, Chen L, Wang J, Kasai T, Kudoh T, Satoh A, Fu L, Seno M (2011). Enhanced internalization of ErbB2 in SK-BR-3 cells with multivalent forms of an artificial ligand. J Cell Mol Med.

[38] Chaudhary VK, Jinno Y, FitzGerald D, Pastan I (1990). Pseudomonas exotoxin contains a specific sequence at the carboxyl terminus that is required for cytotoxicity. Proc Natl Acad Sci U S A.

[39] Jackson ME, Simpson JC, Girod A, Pepperkok R, Roberts LM, Lord JM (1999). The KDEL retrieval system is exploited by Pseudomonas exotoxin A, but not by Shiga-like toxin-1, during retrograde transport from the Golgi complex to the endoplasmic reticulum. J Cell Sci.

[40] Koopmann JO, Albring J, Huter E, Bulbuc N, Spee P, Neefjes J, Hammerling GJ, Momburg F (2000). Export of antigenic peptides from the endoplasmic reticulum intersects with retrograde protein translocation through the Sec61p channel. Immunity.

[41] Melcher A, Gough M, Todryk S, Vile R (1999). Apoptosis or necrosis for tumor immunotherapy: what's in a name?. J Mol Med (Berl).

[42] Decker T, Oelsner M, Kreitman RJ, Salvatore G, Wang QC, Pastan I, Peschel C, Licht T (2004). Induction of caspase-dependent programmed cell death in B-cell chronic lymphocytic leukemia by anti-CD22 immunotoxins. Blood.

[43] Risberg K, Fodstad O, Andersson Y (2009). The melanoma specific 9.2.27PE immunotoxin efficiently kills melanoma cells in vitro. Int J Cancer.

[44] Ribbert T, Thepen T, Tur MK, Fischer R, Huhn M, Barth S (2010). Recombinant, ETA'-based CD64 immunotoxins: improved efficacy by increased valency, both in vitro and in vivo in a chronic cutaneous inflammation model in human CD64 transgenic mice. Br J Dermatol.

[45] Zhu X, Tao K, Li Y, Li S, Zhang L, Wang D, Zhong L, Feng W (2013). A new recombinant immunotoxin hscFv-ETA' demonstrates specific cytotoxicity against chronic myeloid leukemia cells in vitro. Immunol Lett.

[46] Staudinger M, Glorius P, Burger R, Kellner C, Klausz K, Gunther A, Repp R, Klapper W, Gramatzki M, Peipp M (2014). The novel immunotoxin HM1.24-ETA' induces apoptosis in multiple myeloma cells. Blood Cancer J.

[47] Keppler-Hafkemeyer A, Brinkmann U, Pastan I (1998). Role of caspases in immunotoxin-induced apoptosis of cancer cells. Biochemistry.

[48] Keppler-Hafkemeyer A, Kreitman RJ, Pastan I (2000). Apoptosis induced by immunotoxins used in the treatment of hematologic malignancies. Int J Cancer.

[49] Du X, Youle RJ, FitzGerald DJ, Pastan I (2010). Pseudomonas exotoxin A-mediated apoptosis is Bak dependent and preceded by the degradation of Mcl-1. Mol Cell Biol.

[50] Andersson Y, Juell S, Fodstad Ø (2004). Downregulation of the antiapoptotic MCL-1 protein and apoptosis in MA-11 breast cancer cells induced by an anti-epidermal growth factor receptor–Pseudomonas exotoxin a immunotoxin. International Journal of Cancer.

[51] Brinkmann U, Mansfield E, Pastan I (1997). Effects of BCL-2 overexpression on the sensitivity of MCF-7 breast cancer cells to ricin, diphtheria and Pseudomonas toxin and immunotoxins. Apoptosis.

[52] Bogner C, Dechow T, Ringshausen I, Wagner M, Oelsner M, Lutzny G, Licht T, Peschel C, Pastan I, Kreitman RJ, Decker T (2010). Immunotoxin BL22 induces apoptosis in mantle cell lymphoma (MCL) cells dependent on Bcl-2 expression. Br J Haematol.

[53] Yaginuma Y, Westphal H (1992). Abnormal structure and expression of the p53 gene in human ovarian carcinoma cell lines. Cancer Res.

