# Supplementary material for: HER2-specific recombinant immunotoxin 4D5scFv-PE40 passes through retrograde trafficking route and forces cells to enter apoptosis
Source: Oncotarget. 2017 Mar 2;8(13):22048–58. doi: 10.18632/oncotarget.15833 (PMC5400645; doi:10.18632/oncotarget.15833)
Supplement: Supplementary file 1 [file oncotarget-08-22048-s001.pdf]

HER2-specific recombinant immunotoxin 4D5scFv-PE40 passes through retrograde trafficking route and forces cells to enter apoptosis

SUPPLEMENTARY DATA

4D5scFv-PE40 expression and purification

The plasmid for expression of recombinant protein 4D5scFv-PE40 was constructed on the basis of the pSD-4D5-barnase plasmid [Deyev SM et al., Nat Biotechnol. 2003; 21:1486–92]. The DNA fragment encoding PE40 protein was amplified from plasmid pIG6-4D5MOCB-ETA [Di Paolo C et al., Clin Cancer Res. 2003; 9:2837–48] using 5′-actacggcgcgccggagttcccgaaaccgtccac and 5′ -tgcgtaagcttctacagttcgtctttatggtg primers. The product of amplification was cloned into pSD-4D5-barnase plasmid instead of barnase gene using AscI and HindIII endonucleases. The resulting expression cassette consists of an inducible *lac* promoter, OmpA signal peptide coding sequence, 4D5scFv and PE40 genes, hexahistidine tag coding sequence and a KDEL oligopeptide coding sequence on the 3′- terminus. 4D5scFv and PE40 were connected by flexible 16-aa linker from the mouse IgG3 hinge region [Müller KM et al., FEBS Lett. 422 (1998) 259–264]. The resulting construct pSD-4D5scFv-ETA was verified by sequencing.

The 4D5scFv-PE40 protein containing C-terminal His6-tag was expressed in the *E. coli* BL21 strain transformed with pSD-4D5scFv-ETA and grown in lysogeny broth (LB) at 28°C. Expression of 4D5scFv-PE40 was induced by the addition of 0.5 mM IPTG at OD_{550} of 0.8. The bacteria were then incubated at 28°C for 12 h. The cells were harvested by centrifugation, then the pellet was resuspended in the lysis buffer (5 mM Tris-HCl, 40 mM K_2HPO_4, pH 8.3, with 0.5 M NaCl) and sonicated on ice. The lysate was then centrifuged at 22000 g for 30 min at 4°C. The supernatant was used for purification of His6-tagged protein on Ni^{2+}-NTA column (GE Healthcare). The fractions eluted at approximately 250 mM imidazole were used for further purification with ion-exchange chromatography. Elution fractions were diluted 20-fold, applied onto Q Sepharose FF 1-ml column (GE Healthcare) and eluted using linear gradient from 25 to 500 mM NaCl.

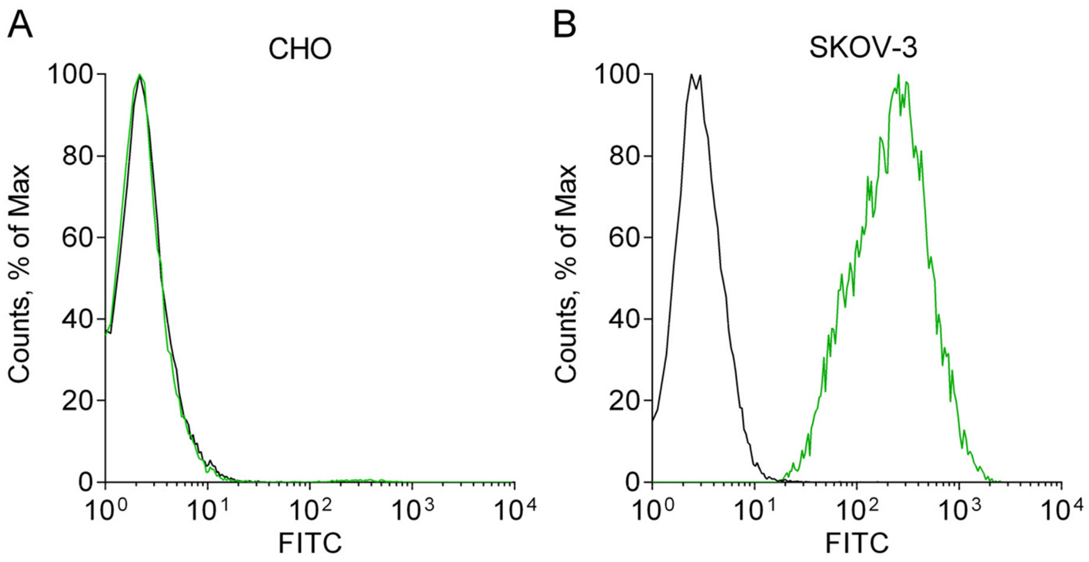

Supplementary Figure 1: Analysis of the level of HER2 expression in cell lines. CHO **(A)** and SKOV-3 **(B)** cells were detached and incubated for 30 min with FITC-labeled mouse monoclonal anti-HER2 antibody (green line) or with FITC-labeled isotypic control (black line) (1 µg per 0.3×10^6 cells in PBS supplemented with 3% *(w/w)* bovine serum albumine). Then cells were washed twice with PBS supplemented with 1% *(w/w)* bovine serum albumine and analyzed by flow cytometry using a FACSCalibur instrument (BD Biosciences) equipped with a 488 nm laser to excite FITC.

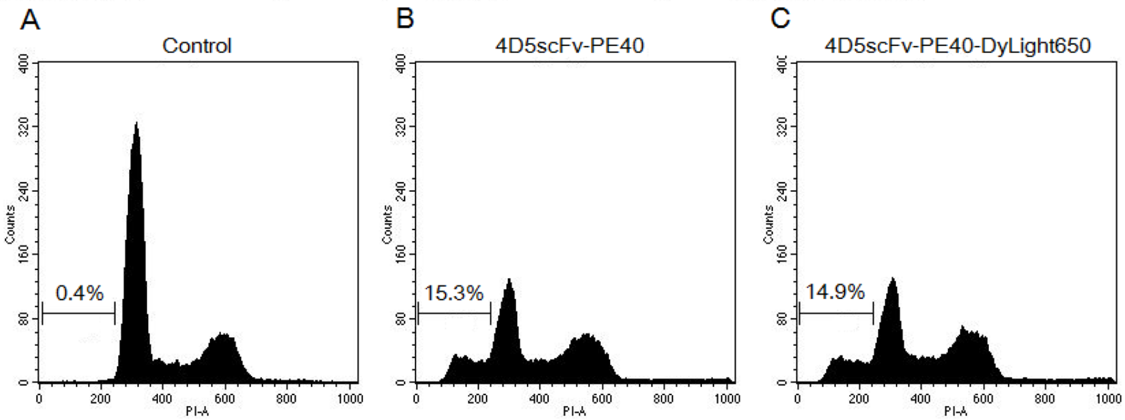

Supplementary Figure 2: Induction of apoptosis in SKOV-3 cells after treatment with 4D5scFv-PE40. Flow cytometry analysis of hypoploid sub-G1 nuclei in control **(A)** and after incubation with 50 nM 4D5scFv-PE40 **(B)** or DyLight650-labeled 4D5scFv-PE40 **(C)** for 72 h.
